# Silencing Quorum Sensing through Extracts of *Melicope lunu-ankenda*

**DOI:** 10.3390/s120404339

**Published:** 2012-03-29

**Authors:** Li Ying Tan, Wai-Fong Yin, Kok-Gan Chan

**Affiliations:** Division of Genetics and Molecular Biology, Institute of Biological Sciences, Faculty of Science, University of Malaya, 50603 Kuala Lumpur, Malaysia; E-Mails: etaly87@yahoo.com (L.Y.T.); yinwaifong@yahoo.com (W.-F.Y.)

**Keywords:** anti-quorum sensing, bioluminescence, *lecA::lux*, Malaysian plants, *N*-acyl-l-homoserine lactones (AHL), *Pseudomonas aeruginosa* PAO1, pyocyanin, virulence, swarming motility

## Abstract

Quorum sensing regulates bacterial virulence determinants, therefore making it an interesting target to attenuate pathogens. In this work, we screened edible, endemic plants in Malaysia for anti-quorum sensing properties. Extracts from *Melicope lunu-ankenda* (Gaertn.) T. G. Hartley, a Malay garden salad, inhibited response of *Chromobacterium violaceum* CV026 to *N*-hexanoylhomoserine lactone, thus interfering with violacein production; reduced bioluminescence expression of *E. coli* [pSB401], disrupted pyocyanin synthesis, swarming motility and expression of *lecA::lux* of *Pseudomonas aeruginosa* PAO1. Although the chemical nature of the anti-QS compounds from *M. lunu-ankenda* is currently unknown, this study proves that endemic Malaysian plants could serve as leads in the search for anti-quorum sensing compounds.

## 1. Introduction

Most Gram-negative bacteria use “quorum sensing” (QS) to coordinate their population behaviour including expression of virulence factors through the action of extracellular signal molecules, such as the *N*-acyl-l-homoserine lactones (AHLs). QS involves coupling of AHLs to a transcriptional activator which in turn modulates QS-mediated gene expressions [1,2]. Gram negative and Gram positive bacteria employ different signal molecules, inasmuch that the former use AHLs while the latter use post-translationally processed peptides [3,4].

*Pseudomonas aeruginosa* is the opportunistic Gram negative bacterium which is a well-studied model for AHL-mediated QS. [5]. *P. aeruginosa* has two individual but interconnected QS systems, namely *las* and *rhl*. Regulation of virulent factors expressed by *P. aeruginosa* is controlled by the *las* and *rhl* [6,7] which are arranged in a hierarchical manner such that the *las* system activates the *rhl* system [7]. Myriad virulence factors of *P. aeruginosa* namely pyocyanin, proteases, haemolysins, exotoxin A and exoenzyme S are QS-dependent [8,9].

Emergence of antibiotic-resistant pathogenic bacteria is now a global threat for public health management. Alternative treatment that does not rely on antibiotics and thus may avoid drug-resistance problems is therefore highly desirable. One such anti-infective treatment is anti-QS molecules, which can quench the virulence phenotypes exerted by pathogenic bacteria [10]. Among the few non bacterial-origin antagonists of QS that have been found are catechin (from *Combretum albiflorum* bark extract), halogenated furanones (from red alga *Delisea pulchra*), raspberry, basil and vanilla extracts [11–14].

Not much reported work has been done on endemic plants in Malaysia that show anti-QS activity although some Malaysian plants have been tested for anti-cancer, anti-diabetic, anti-oxidant and other various assays, but not anti-QS effects [15–17]. Therefore, in this paper, we examined a Malaysian endemic plant, *Melicope lunu-ankenda* (Gaertn.) T. G. Hartley, locally known as “Tenggek burung”, for its anti-QS properties. Leaves of *M. lunu-ankenda* are usually eaten raw as ‘ulam’ (salad) and are traditionally used to revitalize the body as well as to prevent hypertension.

## 2. Experimental Section

### 2.1. Plant Materials and Preparation of Extracts

*M. lunu-ankenda* was obtained from a local market located in Selangor (Malaysia). A voucher specimen of *M. lunu-ankenda* was deposited at the University Malaya Herbarium (Voucher Number: 047697). The plant samples were washed twice with sterile distilled water followed by a final rinse with 70% (v/v) ethanol. Plant samples were dried in an oven at 45 °C for 72 hours. The dried plant samples were ground to a fine powder and submerged sequentially in hexane, chloroform and methanol (ratio 1:10 w/v) for 72 hours. The extracts were filtered through Whatman No.1 paper and concentrated under vacuum using a rotary evaporator. Plant extracts of 10 mg/mL (w/v in 100% DMSO) were diluted with sterile distilled water to 1, 2, 3, 4 and 5 mg/mL prior to use.

### 2.2. Bacterial Strains, Growth Media and Culture Conditions

Bacterial strains used in this study are listed in Table 1. Bacteria were grown in Luria-Bertani (LB) medium (1% w/v NaCl, 1% w/v tryptone, 0.5% w/v yeast extract) with shaking (220 rpm). *Chromobacterium violaceum* CV026 was cultured in 28 °C, while *P. aeruginosa* strains at 37 °C. *C. violaceum* CV026 growth medium was supplemented with kanamycin (30 μg/mL) and chloramphenicol (30 μg/mL).

### 2.3. *C. violaceum* CV026 Assay

*C. violaceum* CV026 assay was performed as described by Renee and Gray [21] with modification. Overnight grown *C. violaceum* CV026 cells (15 mL) were added into 200 mL of molten LB agar that has been supplemented with *N*-hexanoylhomoserine lactone (C6-HSL, 0.25 μg/mL). *C. violaceum* CV026 agar suspension was poured into Petri dishes and allowed to solidify, wells were then made using sterile pipette tips. Plant extract (30 μL) was placed in each well and the extract solvent (DMSO, 50% v/v) served as the negative control. The plates were incubated at 28 °C for 24 hours. Halo formation on a purple background suggested that the plant extracts exhibited anti-QS.

### 2.4. Violacein Quantification Assay

Violacein quantification assay was performed in a 96-well plate [22]. Optical density (OD_600nm_) of overnight culture of *C. violaceum* CV026, supplemented with C6-HSL (0.125 μg/mL), was adjusted to 1.2 prior to use. *C. violaceum* CV026 cells (90 μL) were added to each well followed by the addition of 10 μL of plant crude extract. The 96-well plate was incubated at 28 °C in a shaking incubator. After 16 hours, the mixtures in the 96-well plate were completely dried at 60 °C. DMSO (100 μL) was added onto each well and the microplate was placed in a shaker until all the violacein was solubilized. The absorbance of each well was read at 590 nm using DYNEX MRX Elisa reader (Chantilly, VA, USA).

### 2.5. Quantification of Bioluminescence from *E. coli* [pSB401] and *E. coli* [pSB1075]

Bioluminescence expression was quantified using a Tecan luminometer (Infinite M200, Männerdorf, Switzerland). Briefly, overnight culture of *E. coli* biosensors cells was diluted to an OD_600nm_ of 0.1. Then, 230 μL of *E. coli* biosensors cells and 20 μL of plant extract were added into the well of 96-well microtitre plate. The bioluminescence and OD_495nm_ were determined every 30 min for 24 hours by the luminometer [19]. Expression of bioluminescence was given as relative light unit (RLU)/OD_495nm_ against time [19]. Reduction of bioluminescence in *E. coli* [pSB401] and *E. coli* [pSB1075] suggested anti-QS properties of the plant extracts.

### 2.6. Pyocyanin Quantification Assay

Pyocyanin quantification assay was performed as described by Essar *et al.* [23] with slight modification. Briefly, overnight culture of *P. aeruginosa* PA01 was adjusted to an OD_600nm_ of 0.2. Then, 250 μL of plant extract was added and mixed well with *P. aeruginosa* PA01 cells (4.75 mL) in a polypropylene tube and incubated at 37 °C for 24 hours. The 5 mL culture was extracted with 3 mL of chloroform, followed by mixing the chloroform layer with 1 mL of 0.2 M HCl. The absorbance of the pink extracted organic layer was then measured at 520 nm using the UV-visible spectrophotometer (UV1601, Shidmazu, Kyoto, Japan).

### 2.7. Quantification of *P. aeruginosa* PA01 *lecA* Expression

*P. aeruginosa* PA01 *lecA* expression was quantified using a Tecan luminometer (Infinite M200). Briefly, overnight culture of *P. aeruginosa* PA01 *lecA::lux* was diluted to an OD_600nm_ of 0.1. Then, 230 μL of *P. aeruginosa* PA01 *lecA::lux* and 20 μL of plant extract were added into the well of 96-well microtitre plate. The bioluminescence and OD_495nm_ were determined every 30 min for 24 hours by the Tecan luminometer. *P. aeruginosa* PA01 *lecA::lux* expression was given as relative light unit (RLU)/OD_495nm_ against time [19]. Reduction in the bioluminescence of *P. aeruginosa* PA01 *lecA::lux* suggested anti-QS properties of the plant extracts.

### 2.8. *P. aeruginosa* PA01 Swarming Assay

Swarming agar was prepared by the following compositions: glucose (1% w/v), Bacto agar (0.5% w/v), Bacto peptone (0.5% w/v) and yeast extract (0.2% w/v). Solidified swarming agar (10 mL) was overlaid with 4.75 mL of swarming agar supplemented with 250 μL of plant extract. Overnight culture of *P. aeruginosa* PA01 (2 μL) was inoculated in the centre of the agar and incubated for 16 hours at 37 °C. Reduced swarming motility of *P. aeruginosa* PA01 suggested anti-QS properties of the plant extracts.

### 2.9. Statistical Tests

All assays were performed on triplicate basis and the significance of the data was tested using ANOVA test (*P* < 0.05) using GraphPad Prism software.

## 3. Results and Discussion

The purpose of this study was to investigate the anti-QS properties of endemic Malaysian plants, in particular, edible plants. *M. lunu-ankenda* is a kind of “ulam” that is widely consumed as garden salad by the Malay community in Malaysia and it has shown no adverse effects on human health. It is also traditionally used to treat hypertension. However, not much scientific studies have been carried out on *M. lunu-ankenda* and there is no reported work that studies the anti-QS properties of *M. lunu-ankenda*.

### 3.1. CV026 Plate Assay

**Figure 1.**
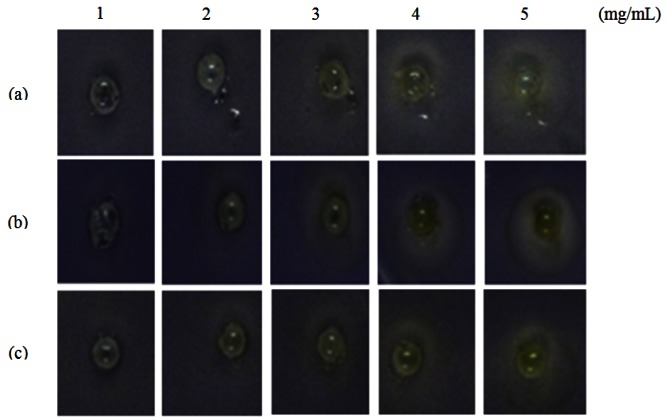
Figure shows each well containing 30 μL of *M. lunu-ankenda* extracted with the following solvents: (**a**) hexane; (**b**) chloroform; (**c**) methanol, applied at the concentration of 1, 2, 3, 4 and 5 mg/mL.

Formation of a visible halo zone indicates anti-QS action exerted by the plant extract. In Figure 1, a halo zone was formed when the extract was applied at 1 mg/mL and the size of the halo zone increased in parallel with the concentration of the *M. lunu-ankenda* hexane extract (Figure 1).

This suggests stronger anti-QS action of the extract corresponds to its increasing concentration. In contrast, *M. lunu-ankenda* chloroform extract showed weaker anti-QS properties as it only started to exert its anti-QS effect at a concentration of 2 mg/mL (Figure 1). As compared to the *M. lunu-ankenda* hexane extract, the methanolic extract showed weaker anti-QS activity (Figure 1). DMSO (50% v/v) served as the negative control which showed no bactericidal or anti-QS effects (data not shown).

### 3.2. Violacein Quantification Assay

**Figure 2.**
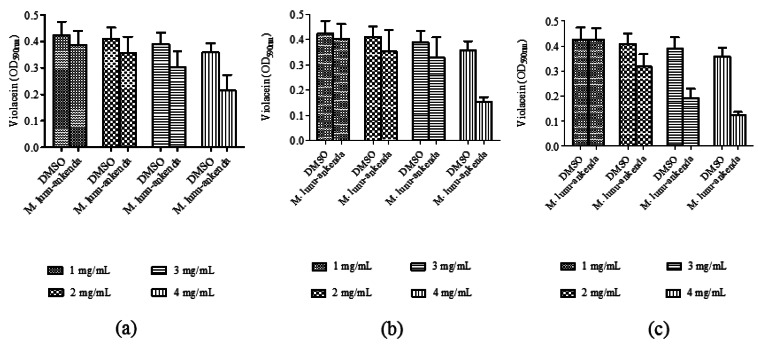
Inhibition of violacein production by *M. lunu-ankenda* extracted with the following solvents: (**a**) hexane; (**b**) chloroform and (**c**) methanol.

Anti-QS activity of *M. lunu-ankenda* extracts (applied at 1, 2, 3 and 4 mg/mL) were analyzed using violacein quantitative assay and DMSO (10%, 20%, 30% and 40% v/v) served as the negative controls at each corresponding concentrations. By using the statistical ANOVA test, it was found that *M. lunu-ankenda* chloroform and methanol extracts of 4 mg/mL causes significant inhibition of violacein as compared to the control (Figure 2). In general, there was reduction in violacein production as the concentration of the extracts increases. However, these reductions were not significantly different at *P* < 0.05.

*C. violaceum* is a Gram negative bacterium which synthesizes the purple pigment violacein, a QS-mediated trait regulated by C6-HSL. *C. violaceum* CV026, on the other hand, is a transposon mutant strain of *C. violaceum* that is unable to synthesize C6-HSL. Thus, *C. violaceum* CV026 can only produce violacein in the presence of exogenous short chain AHLs [14,18]. In *C. violaceum* CV026 plate assay, formation of halo zone indicates the plant samples is either inhibiting the C6-HSL competitively from binding to its transcriptional regulator, cviR; degrading the C6-HSL enzymatically, or removing the C6-HSL via active transport [24–26]. Inhibition on violacein production was quantified and from the results obtained in this study, it was proven that *M. lunu-ankenda* reduced violacein production significantly. In agreement to this finding, other plant extracts such as vanilla, *Tremella fuciformis, Conocarpus erectus, Quercus virgiana*, pea seedlings and other various higher plants have been found to possess anti-QS activity against biosensor strain *C. violaceum* CV026 [10,14,24,27,28].

### 3.3. Quantification of Bioluminescence from *E. coli* pSB[401] and *E. coli* pSB[1075]

All three solvents extracts of *Melicope lunu-ankenda* showed significant inhibition against *E. coli* [pSB401], but not *E. coli* [pSB1075] (data not shown). *E. coli* [pSB1075] produces optimum luminescence in the presence of exogenous *N*-3-dodecanoylhomoserine lactone (3-oxo-C12-HSL) [20]. Plant extracts that reduced bioluminescence of *E. coli* [pSB401] and *E. coli* [pSB1075] suggest the presence of anti-QS activity.

### 3.4. Pyocyanin Quantification Assay

**Figure 3.**
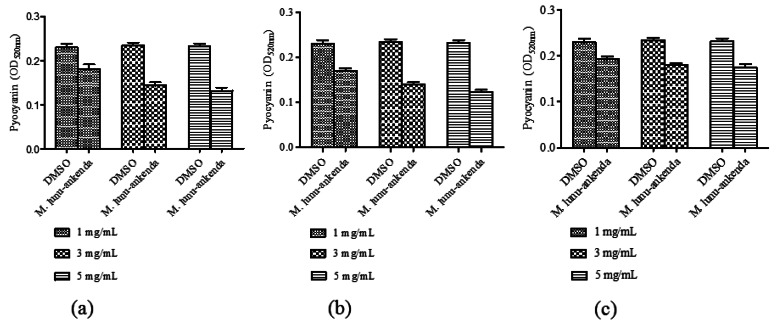
Inhibition of pyocyanin production by *M. lunu-ankenda* extracted with the following solvents: (**a**) hexane; (**b**) chloroform and (**c**) methanol.

Pyocyanin is one of the exoproducts produced by *P. aeruginosa*. It causes extensive cellular damage in the lungs of cystic fibrosis patients. Chloroform and hexane extracts of *M. lunu-ankenda* showed significant reduction in pyocyanin production (Figure 3(a,b)). The methanolic extract only showed significant reduction of pyocyanin at 3 mg/mL (Figure 3(c)).

Pyocyanin can be found in great quantity in the sputum of cystic fibrosis patients and it is also highly permeable to the biological membranes. *P. aeruginosa* causes developing loss of pulmonary function which leads to premature death in the majority of cystic fibrosis patient [29,30]. Mutations in *lasR-lasI, rhlR-rhlI* and the *mvfR-haq* QS systems caused loss in pyocyanin production [31,32]. These QS systems are also involved in production of among others, rhamnolipids, proteases and elastase [30]. Pyocyanin formation involved a multifactorial system and though extracts of *M. lunu-ankenda* could significantly inhibit its production in this study, it is possible that the compounds from *M. lunu-ankenda* influence directly the virulence factor production in a QS independent manner. A study found that cyclic disulphides and trisulphides obtained from garlic have no antibiotic properties but instead suppress the expression of LuxR and LuxR based QSI in *P. aeruginosa* [33,34].

### 3.5. Quantification of *P. aeruginosa PA01 lecA* Expression

**Figure 4.**
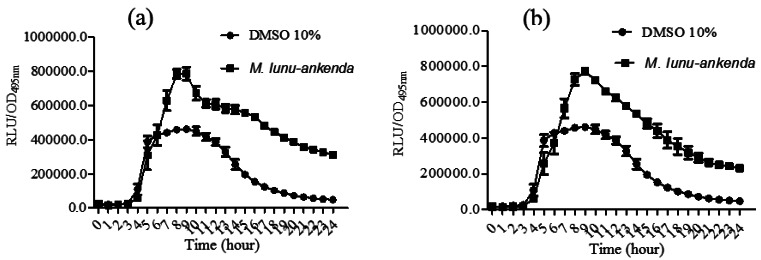
*P. aeruginosa* PA01 *lecA* expression after treated by *M. lunu-ankenda* extracted with (**a**) hexane; (**b**) chloroform, all applied at 1 mg/mL.

*P. aeruginosa* PA01 *lecA::lux* was constructed by the cloning of *luxCDABE* (from *Photorhabdus luminescens*) into the *lecA* gene region of *P. aeruginosa* [19,20]. The hexane and chloroform extracts of *M. lunu-ankenda* caused a significant reduction in PA01 *lecA* expression (Figure 4). However, the methanolic extract did not show any observable results (data not shown). *lecA* is the structural gene of PA-IL, which is a cytotoxic lectin. Synthesis of lectin is directly related to the *rhl* locus as it has been demonstrated that in the *lasR* mutant, lectin synthesis was delayed but not prevented totally [19,35,36]. Reduction of *P. aeruginosa* PA01 *lecA::lux* expression by *M. lunu-ankenda* extracts indicates that these extracts were exerting theirs activity more on the *rhl* system which also controls the expression of elastase and biofilm. Similarly, rosmarinic acid reduces the expression of proteases, elastases and biofilm in *P. aeruginosa* has been reported [37].

### 3.6. Swarming Assay

**Figure 5.**
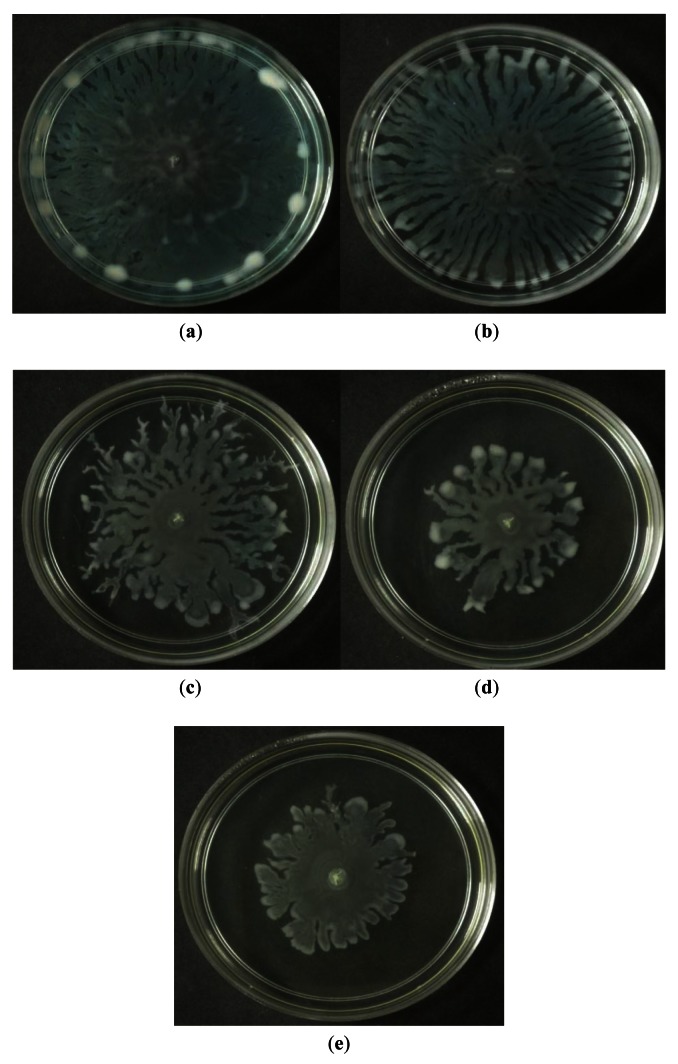
Swarming inhibition assays. Swarming agars of (**a**) *P. aeruginosa* PAO1 supplemented with (**b**) DMSO 30% (v/v, negative control); and *M. lunu-ankenda*-chloroform extracts of (**c**) 1 mg/mL (**d**) 2 mg/mL and (**e**) 3 mg/mL. Images shown are *P. aeruginosa* PAO1 swarming patterns and inhibition effects after 16 hours of incubation in 37 °C.

Swarming refers to the bacterial surface translocation which is QS-dependent and it requires flagella and pili [38,39]. Of the three extracts of *M. lunu-ankenda*, only the chloroform extract showed inhibition against the swarming of *P. aeruginosa*. Swarming, swimming, gliding, twitching, sliding and darting are modes of surface translocation used by bacteria [38]. In this study, we investigated the anti-swarming ability of *M. lunu-ankenda* extract against *P. aeruginosa* PAO1. Generally, there are 3 stages in the swarming process, firstly, differentiation of vegetative cells into swarmer cells, followed by migration of swarmer cell populations and finally consolidation [40,41]. The extract of *M. lunu-ankenda* were seeded into the swarming agar and as shown in Figure 5, the chloroform extract showed observable inhibition against the swarming of *P. aeruginosa* PAO1. The extract of *M. lunu-ankenda* might be exerting its inhibition of swarming during the migration of swarmer cells or causing biofilm dispersal. Similar observation has also been observed in malabaricone C, a compound purified from nut meg extract [42]. Subsequently, as the migration process is a QS-regulated trait, further studies can be done by using the mutants of PA01 which is deficient in QS.

## 4. Conclusions

*M. lunu-ankenda* shows promising anti-QS properties and it has been confirmed that its extracts inhibited QS-dependent virulence determinants of the human pathogens namely *P. aeruginosa* PAO1. The anti-QS compound from *M. lunu-ankenda* may be a new class of non-bacterial origin antagonist. We are currently in the process of fractionating the *M. lunu-ankenda* extracts by using column chromatography. Identification of the *M. lunu-ankenda* anti-QS compound is of great interest because it might be possible to overcome the problems posed by emerging antibiotic-resistant bacteria. The promising results obtained from this study prompt us to investigate more Malaysians' endemic plants.

## Figures and Tables

**Table 1.** Strains Used in This Study.

**Strain**	**Description**	**Source/Reference**

*C. violaceum* CV026	Double mini-Tn*5* mutant derived from ATCC 31532, Kan^R^, Hg^R^, *cvil*::Tn*5 xyl*E, plus spontaneous Str^R^ AHL biosensor, produces violacein pigment only in the presence of exogenous AHL	[18]

*P. aeruginosa* PA01 *lecA::lux*	Prototroph *lecA::luxCDABE* genomic reporter fusion in PA01	[19]

*Escherichia coli*		
[pSB401]	*luxRluxl*' (*Photobacterium fischeri* [ATCC 7744])::*luxCDABE* (*Photorhabdus luminescens* [ATCC 29999]) fusion; pACYC184-derived, Tet^R^, AHL biosensor producing bioluminescence	[20]
[pSB1075]	*lasRlasl*'(*P. aeruginosa* PAO1)::*luxCDABE* (*Photorhabdus luminescens* [ATCC 29999]) fusion in pUC18 Amp^R^, AHL biosensor producing bioluminescence	[20]
